# Size-selective downstream processing of virus particles and non-enveloped virus-like particles

**DOI:** 10.3389/fbioe.2023.1192050

**Published:** 2023-05-25

**Authors:** Nils Hillebrandt, Jürgen Hubbuch

**Affiliations:** Institute of Engineering in Life Sciences, Section IV: Biomolecular Separation Engineering, Karlsruhe Institute of Technology (KIT), Karlsruhe, Germany

**Keywords:** virus particles, virus-like particles, vaccines, downstream processing, purification, size-selective

## Abstract

Non-enveloped virus-like particles (VLPs) are versatile protein nanoparticles with great potential for biopharmaceutical applications. However, conventional protein downstream processing (DSP) and platform processes are often not easily applicable due to the large size of VLPs and virus particles (VPs) in general. The application of size-selective separation techniques offers to exploit the size difference between VPs and common host-cell impurities. Moreover, size-selective separation techniques offer the potential for wide applicability across different VPs. In this work, basic principles and applications of size-selective separation techniques are reviewed to highlight their potential in DSP of VPs. Finally, specific DSP steps for non-enveloped VLPs and their subunits are reviewed as well as the potential applications and benefits of size-selective separation techniques are shown.

## 1 Introduction

Virus-like particles (VLPs) are multimeric structures that resemble viruses but lack the viral genome which makes them non-infectious (Chackerian, 2007). The dense and repetitive structure of antigens leads to a high immunogenicity of VLPs. The high immunogenicity in combination with their non-infectivity makes VLPs potent vaccine candidates (Chackerian, 2007). Non-enveloped VLPs consist of at least one structural protein, several of which assemble into capsids of one or more layers (Roy & Noad, 2009). Non-enveloped VLP-based vaccines licensed for human use protect against hepatitis B virus, human papillomavirus (HPV), hepatitis E virus, and one chimeric VLP-based vaccine protects against malaria (Lua et al., 2014; Nooraei et al., 2021). Another application of non-enveloped VLPs is their utilization as viral vectors for the delivery of nucleic acids, peptides, or drugs (Garcea & Gissmann, 2004; Le & Müller, 2021). In the case of enveloped VLPs, an additional lipid membrane layer is formed by the budding of capsids from the host cell (Fuenmayor et al., 2017). Enveloped VLPs can also consist of a layer of host cell lipid membrane containing viral proteins (Lua et al., 2014). The insertion of antigens or epitopes into the subunit proteins (of non-enveloped and enveloped VLPs), for example by genetic fusion or chemical conjugation, leads to chimeric VLPs (Grgacic & Anderson, 2006; Roldão et al., 2010). Chimeric VLPs were developed as prophylactic and therapeutic vaccines against several diseases including infectious diseases and cancers (Roldão et al., 2010; Mohsen et al., 2020). Illustrations and further information on the different VLP types can be found in Lua et al. (2014).

VLPs can be produced in bacteria, yeast, insect, plant, or mammalian cells (Kushnir et al., 2012; Fuenmayor et al., 2017). VLP properties influence the choice of the host cell for recombinant production of VLPs, for example, eukaryotic cells for lipid envelopes or post-translational protein modifications. Enveloped VLPs are released from cells by budding while viral proteins may also be released by cell lysis. Non-enveloped VLPs can spontaneously self-assemble in host cells (*in vivo*), even in prokaryotic ones such as *Escherichia coli (E. coli)* (Kushnir et al., 2012). Therefore, non-enveloped VLPs are often produced in microbial cells which usually requires cell lysis, such as for hepatitis B core antigen (HBcAg) in *E. coli* (Schumacher et al., 2018) or HPV in *Saccharomyces cerevisiae* (Cook et al., 1999). The necessity of a lysis step or low cell viability leads to a release of host cell impurities into the VLP-containing process solution impacting the downstream processing (DSP) (Moleirinho et al., 2020).

**FIGURE 1:**
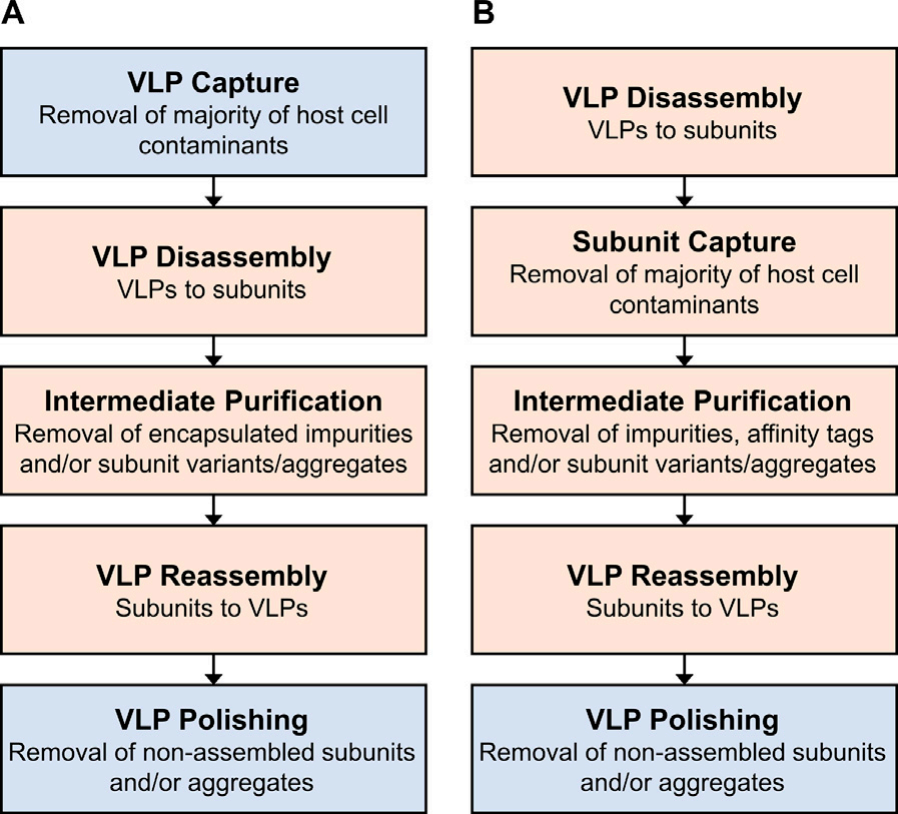
Overview of purification steps and approaches for non-enveloped VLPs. **(A)** Initial purification of assembled VLPs followed by dis- and reassembly. **(B)** Purification of VLP subunits followed by reassembly. The purification techniques presented in Section 2 and Section 3 can be applied in the process steps highlighted in blue and orange, respectively. Note, depending on the impurity profile and purity demands, process steps might require more than one unit operation.

DSP is divided into recovery and purification in this review. The recovery of non-enveloped VLPs is similar to the one in conventional protein DSP. The purification of non-enveloped VLPs can be subdivided into two parts (Pattenden et al., 2005). The first part is the purification of the particles as a whole by removing process-related impurities, for example host cell impurities. This initial purification is referred to as VLP capture and shown as the first step in Figure 1A. It is similar to or overlapping with the purification of non-enveloped viruses and to some extent also with enveloped VLPs and viruses. These different virus-based or viral particles are referred to as virus particles (VPs) in this review. The second part of purification is more specific to non-enveloped VLPs and is optional depending on the VLP and its application. It aims to improve the particle quality since the *in vivo* assembly of VLPs may lead to malformed particles (Roldão et al., 2012), thus product-related impurities. The *in vitro* disassembly of VLPs into subunit proteins and subsequent reassembly (Figure 1A) were shown to improve VLP properties such as immunogenicity and stability (Mach et al., 2006; Zhao, et al., 2012a; Zhao, et al., 2012b). Furthermore, this process sequence enables to remove entrapped impurities (Vicente, et al., 2011a; Link et al., 2012; Strods et al., 2015; Mohsen et al., 2018). For gene therapy applications, bound nucleic acids need to be removed to ensure product safety and free binding sites for target nucleic acids (Strods et al., 2015; Petrovskis et al., 2021). Another approach is performing purification of VLP subunits in the disassembled state, followed by *in vitro* assembly (Liew et al., 2012; Gerstweiler et al., 2021) as shown in Figure 1B. This approach offers the possibility to use purification techniques applied in conventional protein purification without adaptions due to the particulate structure of VLPs. However, prevention of self-assembly during processing is required, for example by supplementing reducing, chelating, or chaotropic agents. The presence of these substances may impact or limit the unit operations available. Furthermore, VLPs consisting of different subunit proteins would require multiple downstream processes. This processing route also prevents exploiting the size difference between particles and impurities during purification (Morenweiser, 2005). Using size-selective separation techniques offers the possibility to process different variants of a VP or VLP subunit similarly. For example, different virus strains, chimeric VLP candidates, or therapeutic cargo within the particle could be purified using the same platform process. However, compared to monoclonal antibodies, platform processes for VPs are not well established (Moleirinho et al., 2020).

## 2 Virus particles

This section focuses on the removal of process-related impurities which is usually performed early in DSP. Typical process-related impurities resulting from host cells are proteins, nucleic acids, and cell debris. Product-related impurities are aggregated VPs, fragmented VPs, or empty viral vectors (Gagnon, 2009). Co-packaged host cell deoxyribonucleic acid (DNA) and non-infectious or empty particles are considered product-related impurities in the case of viral vectors (European Medicines Agency Committee for Advanced Therapies CAT, 2018). Further considerations for product-related impurities of non-enveloped VLPs are reviewed in Section 3.

Table 1 lists recently published DSP approaches for a variety of VPs. It provides an overview of state-of-the-art strategies with the potential for DSP of non-enveloped VLP, even though not all listed VPs are non-enveloped VPs or VLPs. The capture step, at the beginning of the purification train, aims to remove most of the process-related impurities. For chromatographic separation, highly selective affinity ligands are available, for example, protein A for monoclonal antibodies. However, for VPs, the number of commercially available affinity ligands is small or non-existent requiring costly and custom-made solutions. Alternatives for VPs are separations based on size promising universal processing independent of strain, construct, or candidate. Additionally, size-selective separation is beneficial due to the large size differences between VPs and common impurities. This is also reflected in Table 1 in which the size-selective separation techniques filtration, steric exclusion chromatography (SXC), size-exclusion chromatography (SEC), multimodal SEC (mmSEC), and precipitation are frequently used. These techniques are highlighted in the following paragraphs.

**TABLE 1:** Examples of recently published downstream processes (recovery and purification with step yield in parentheses when available) for VPs. If the recovery steps do not contain a lysis step, culture supernatants were processed.

VP	Type	Host cell	Recovery	Purification	Reference
Adenovirus	Non-enveloped	A549	Lysis	UF/DF (92%)	Moleirinho et al. (2018)
			Nuclease	AEX (72%)	
			MF_d_ (60%)	UF/DF (79%)	
			MF_d_ (94%)	SEC (56%)	
HBcAg VLP	Non-enveloped	*E. coli*	Lysis	AMS precipitation	Zhang et al. (2021)
			Centrifug.	Disassembly (89%)	
				Nuclease	
				imAC	
				Reassembly	
Hepatitis C virus	Enveloped	Huh7.5	MF_d_ (∼100%)	UF (45%)	Lothert, et al. (2020a)
			MF_d_ (∼100%)	Inactivation	
				Nuclease	
				SXC_m_ (99%)	
				psAC_m_ (50%)	
Influenza A virus	Enveloped	MDCK	MF_m_	SXC_m_ (≥100%)	Bissinger et al. (2021)
			Nuclease	psAC_m_ (84%)	
			Inactivation		
			MF_m_		
Influenza A VLP	Enveloped	High five	Nuclease	UF/DF (94%)	Carvalho et al. (2019)
			MF_d_	UF/DF (81%)	
			MF_m_		
Lentiviral vector	Enveloped	HEK293	Nuclease	UF/DF (≥100%)	Valkama et al. (2020)
			MF_d_ (≥100%)	AEX_m_ (22%)	
Measles virus vector	Enveloped	Vero	MF_d_ (≥100%)	Nuclease (≥100%)	Steppert et al. (2022)
				mmSEC (87%)	
				UF/DF (85%)	
Orf virus	Enveloped	Vero	Lysis	Nuclease (∼100%)	Lothert, et al. (2020b)
			MF_d_ (91%)	SXC_m_ (92%^a^)	
			MF_d_ (77%)	mmSEC (97%^a^)	
Adeno-associated virus	Non-enveloped	HEK293	MF_m_	UF (59%^a^)	Tomono et al. (2018)
				Nuclease	
				Heat precipitation	
				AMS precipitation	
				AEX (63%^a^)	
				UF	
				SEC (87%^a^)	
Zika/Yellow fever VLP	Enveloped	HEK293	MF_m_ (82%^a^)	AEX (91%^a^)	Lima et al. (2019)
				mmSEC (90%^a^)	

Abbreviations: AMS, ammonium sulfate; centrifug, centrifugation; imAC, immobilized metal affinity chromatography; MF, microfiltration; psAC, pseudo affinity chromatography. For abbreviations of the host cells, refer to the respective reference. Superscripts: a, step yield was averaged over studied VP variants. Subscripts: d, depth filtration; m, membrane (filtration).

Filtration, such as (cross-flow) microfiltration and cross-flow ultrafiltration/diafiltration (UF/DF), is commonly used during DSP of VPs as indicated by Wolff & Reichl (2011) and in Table 1. Microfiltration, mostly in normal-flow mode, is used for clarification during recovery whereas both cross-flow mirco- and UF/DF are used during purification. VPs are either retained by the membrane and smaller impurities (for example host cell proteins and DNA) are depleted or larger particles (for example cell debris or aggregates) are retained and VPs permeate through the membrane (Wickramasinghe et al., 2005; Grzenia et al., 2008). Cross-flow UF/DF has several advantages compared to other commonly used purification techniques for VPs. The small pore size of UF membranes makes them an alternative to ultracentrifugation (Reiser, 2000; Peixoto et al., 2007). Compared to UF/DF, ultracentrifugation, both density gradient and continuous, is generally considered to be poorly scalable, costly, time-consuming, and requires an additional buffer exchange in case of density gradients (Morenweiser, 2005; Vicente, et al., 2011b; Wolff & Reichl, 2011; Ladd Effio & Hubbuch, 2015; Nestola et al., 2015; Moleirinho et al., 2020). Carvalho et al. (2019) showed that the purification of Influenza A VLPs using two UF/DF steps is more efficient in terms of process duration and buffer consumption when compared to ion exchange chromatography and SEC, however with the drawback of low DNA removal. The use of UF/DF during purification offers potential integration of a formulation step which can also be performed by UF/DF (Liew et al., 2012; Wagner et al., 2014; Carvalho et al., 2019; Valkama et al., 2020; Moreira et al., 2021).

SEC separates molecules according to their ability to penetrate the pores of porous resin beads within a column. Therefore, larger particles are excluded from either all or some of the pores depending on the pore and particle size. With decreasing size, solutes diffuse into an increasing fraction of the pores leading to a longer residence time in the column. SEC thus has a high selectivity for the separation of VPs and host cell impurities, and is mostly independent of the liquid phase conditions (Gagnon, 2009). SEC with pore sizes that exclude VPs enables their elution in the void volume of the column while impurities elute later. This enables higher flow rates, shorter columns, and higher loads, reducing some of the drawbacks in conventional protein SEC (Gagnon, 2009). However, SEC leads to dilution and is limited by restricted loading volumes, especially for separation problems with similar size magnitudes, such as VPs and their aggregates. SEC thus benefits from reduced process volumes and is therefore often performed toward the end of purification as shown in Table 1 and other studies (Peixoto et al., 2007; Rodrigues et al., 2007; Tomono et al., 2016). Similarly to UF/DF mentioned above, the polishing of VPs by SEC is often performed in combination with a formulation step (Peixoto et al., 2007; Dormond et al., 2010; Merten et al., 2011; Moleirinho et al., 2018).

Next to SEC as a conventional chromatographic technique for VP purification (Gagnon, 2009), modern mmSEC (commercially available as Capto Core 400/700 by Cytiva) is increasingly applied for purification in recent publications [Table 1 and (Weigel et al., 2014; Reiter et al., 2019)]. mmSEC is based on the core-shell bead technology where the core with multimodal ligands is surrounded by an inert size-restricting shell. This technology enables the binding of smaller impurities and recovery VPs in the flow-through. The drawback of mmSEC is that the required capacity is determined by the impurity content, not the target species content. This property makes it more attractive in later process stages with a lower impurity burden, for example during polishing.

Another chromatographic technique that is beneficial for the purification of VPs and larger proteins is SXC (J. Lee et al., 2012). Here, the addition of polyethylene glycol (PEG) to the VP solution increases the free energy of the system. The increase in free energy is attributed to the steric exclusion of PEG molecules from the VP surface and other surfaces leading to an energetically unfavorable discontinuity in PEG concentration. This excluded volume effect is highly correlated to the VP or protein hydrodynamic radius but is also affected by other solute and solvent properties (J. Lee et al., 2012). The association of VPs with each other and hydrophilic surfaces reduces the free energy, which is thermodynamically more favorable. Using monolith columns (J. Lee et al., 2012) or membranes (Marichal-Gallardo et al., 2017) as stationary phases allows for the binding and elution of VPs by increasing and decreasing the PEG concentration in the mobile phase, respectively. In addition to the examples in Table 1, SXC is applied as a capture step for adeno-associated viruses achieving a high recovery (Marichal-Gallardo et al., 2021). The advantages of SXC are binding and elution under practically any conditions only requiring the addition and removal of PEG, respectively, as well as low costs and good scalability using membranes (Marichal-Gallardo et al., 2017).

Purification of VPs using PEG precipitation underlies similar principles as SXC (J. Lee et al., 2012). As for SXC, larger proteins or particles tend to precipitate earlier than smaller species at identical conditions (Rothstein, 1994). Besides using non-ionic polymers such as PEG, protein precipitation is performed by increasing the salt concentration (salting-out), for example using ammonium sulfate as a precipitant (Wingfield, 1998). Salting-out is dominated by a preferential exclusion of the precipitant from the hydrated protein surface leading to self-association and precipitation (Timasheff & Arakawa, 1988; Wingfield, 1998). Precipitation is usually applied for capturing at the beginning of purification. Examples are the precipitation of norovirus VLPs using PEG (Koho et al., 2012) or precipitation of different HBcAg VLP candidates (Schumacher et al., 2018; Zhang et al., 2021) and adeno-associated virus strains using ammonium sulfate (Tomono et al., 2016; 2018). For the screening of VLP precipitation conditions, advanced analytical and predictive tools were recently developed (Vormittag et al., 2020; Wegner & Hubbuch, 2022). Integration of precipitation, wash, and re-dissolution with cross-flow filtration led to synergies improving process performance (Hillebrandt et al., 2020).

### 2.1 Discussion of non-size-selective separation techniques

A benchmark for host cell DNA of continuous cell lines according to the European Pharmacopoeia is a reduction to a maximum of 10 ng per vaccine dose to minimize tumorigenic potential (Council of Europe, 2017). The reduction of host cell nucleic acids in DSP of VPs is often performed enzymatically by nucleases at the end of upstream processing or early in DSP. Nuclease application is present in almost all processes listed in Table 1 and in older downstream processes such as for HPV VLPs (Cook et al., 1999). However, nucleases require specific conditions for optimal activity and thus temperature, pH, or ion composition may have to be adapted. Especially for high nucleic acid burden after cell lysis, efficient processing may require a trade-off between incubation time and product loss due to instability at the chosen conditions (Kawka et al., 2021). However, also short processing within 30 min, at room temperature, and without product loss was demonstrated (Lothert, et al., 2020a). Nevertheless, even for long incubation times, nucleic acid removal was observed to be incomplete (Weigel et al., 2014; Lothert, et al., 2020b; Kawka et al., 2021). At a large production scale, the use of nuclease and required conditioning steps may considerably increase production costs. Nuclease removal during further DSP is another important consideration for patient safety. For gene therapy applications, the removal of nucleases is a crucial step as it prevents the digestion of target nucleic acids.

Apart from size-selective separations, heparin and sulfated cellulose are applied as adsorbers for (pseudo) affinity chromatography. Both have a similar molecular structure and were shown to preferentially bind certain VPs (Opitz et al., 2007; 2009). Heparin ligands were applied for the separation of human immunodeficiency virus-1 gag VLPs and extracellular vesicles (Reiter et al., 2019) as well as HPV VLP purification (Kim et al., 2010). Sulfated cellulose membrane adsorbers were used for pseudo affinity capture of influenza virus (Opitz et al., 2009; Weigel et al., 2016) and VLPs (Carvalho et al., 2018) as well as for polishing of hepatitis C and influenza virus (Table 1). Further chromatography types are also used where anion exchange chromatography (AEX) is often applied for purification of enveloped (Table 1) and non-enveloped VPs such as adenovirus (Kawka et al., 2021), norovirus VLPs (Koho et al., 2012), and HPV VLPs (Cook et al., 1999). A potential disadvantage of ion exchange chromatography is the elution at a high ionic strength or a different pH, which might lead to VP aggregation or instability and thus requires optimization for each new strain or candidate. Overall, bead-based bind-elute chromatography of VPs, such as ion-exchange or affinity, often suffers from low dynamic binding capacities due to size-exclusion and low diffusion coefficients of VPs (Gagnon, 2009). These disadvantages can be mitigated using stationary phases for which convective mass transfer of VPs to the ligands is dominant, for example membranes or monoliths (Gagnon, 2009).

## 3 Non-enveloped VLPs

As described above, non-enveloped VLPs can be produced as subunits, purified, and assembled (Figure 1B) or first purified as a whole, optionally disassembled into subunits, and subsequently reassembled (Figure 1A). With regards to the latter approach, Section 2 elaborates on the purification of VPs as a whole which enables to exploit their size for purification. This section reviews subsequent purification steps including VLP dis- and reassembly which are specific for non-enveloped VLP and their subunits. For each process step, one or more size-selective purification approaches are presented.

The disassembly of non-enveloped VLPs can be achieved by changing the liquid phase conditions, for example by the addition of dithiothreitol in combination with a pH increase for HPV VLPs (Mach et al., 2006), the addition of urea or guanidine hydrochloride at low ionic strength for HBcAg VLPs (K. W. Lee & Tan, 2008; Singh & Zlotnick, 2003), or a pH increase at low ionic strength for Norwalk VLPs (Ausar et al., 2006). Liquid phase conditions for disassembly at a laboratory scale are achieved by the direct addition of substances (Zlotnick et al., 1996), by the addition of stock solutions (Hillebrandt et al., 2021; K. W; Lee & Tan, 2008; Valentic et al., 2022), or by dialysis (Ausar et al., 2006; Porterfield et al., 2010; Holmes et al., 2015; Strods et al., 2015). At a larger scale, DF was shown to achieve efficient disassembly processing and purification while avoiding target protein loss due to local urea concentration or pH peaks (Hillebrandt et al., 2021). Another option is pelleting the target protein in form of inclusion bodies (Suffian et al., 2017) or by precipitation (Zhang et al., 2021) with subsequent re-solubilization at disassembly conditions. However, pelleting the target protein by centrifugation was shown to entrap impurities and leads to longer re-solubilization times due to floc compaction when compared to DF-based processing (Hammerschmidt et al., 2016; Hillebrandt et al., 2020).

At a laboratory scale, the purification of the disassembled VLP subunits is often performed by SEC (Strods et al., 2015; Schumacher et al., 2018) or poly-histidine tags with corresponding affinity chromatography (Middelberg et al., 2011; Zhang et al., 2021). However, SEC has limited scalability and affinity tags may lead to undesired alterations in the protein structure or require subsequent removal of the tag. Alternatively, disassembled VLPs were separated from nucleic acids by cross-flow DF and from higher molecular weight species by dead-end UF (Hillebrandt et al., 2021). Other (chromatographic) separations, for example ion exchange, are also conceivable, when compatible with disassembly agents as mentioned above. Strods et al. (2015) suggested and applied alkaline hydrolysis of nucleic acid impurities which, however, requires sufficient stability of the subunit proteins.

Reassembly is usually initiated by reversing the disassembly conditions, hence by increasing ionic strength, decreasing pH to the neutral range, or removing chaotropic or reducing agents (Wingfield et al., 1995; Mach et al., 2006; Ren et al., 2006; Porterfield et al., 2010; Zhang et al., 2021). For gene therapy applications, reassembly can be induced by the addition of nucleic acids which are subsequently encapsulated similarly as in natural virus assembly (Porterfield et al., 2010; Strods et al., 2015; Petrovskis et al., 2021). It is worth mentioning, that different molecular variants of a VLP show different dis- and (re-) assembly behavior, which also affects process development (Rüdt et al., 2019; Hillebrandt et al., 2021; Valentic et al., 2022). VLP dis- and reassembly were investigated in the frame of DF providing a scalable process and the opportunity to improve VLP yield by controlled buffer exchange (Liew et al., 2012; Hillebrandt et al., 2021). Furthermore, process monitoring approaches were developed to monitor product and process properties during DF-based VLP dis- and reassembly (Rüdt et al., 2019; Hillebrandt et al., 2022).

## 4 Conclusion

This review summarizes the fundamentals and applications of several different size-selective separation techniques for the DSP of VPs. Recently published processing routes and advantages of size-selective separation for VPs and non-enveloped VLPs are highlighted. In the future, the presented size-selective approaches can serve as building blocks for platform downstream processes. A combination of different size-selective techniques may lead to synergistic effects in process performance while promising wide applicability.

## Abbreviations

AEX, anion exchange chromatography; DF, diafiltration; DNA, deoxyribonucleic acid; DSP, downstream processing; *E. coli*, *Escherichia coli*; HBcAg, hepatitis B core antigen; HPV, human papillomavirus; mmSEC, multimodal size-exclusion chromatography; PEG, polyethylene glycol; SEC, size-exclusion chromatography; SXC. steric exclusion chromatography; UF, ultrafiltration; VLP, virus-like particle; VP, virus particle.
